# Health Tract, No. 257

**Published:** 1866-11

**Authors:** 


### HEALTH TRACT, No. 257.

### PICKLES.

THERE is a general impression that Pickles are extremely unhealthful; the truth is, they are more easily digested than any other codiment, as they are a pleasant medium of conveying an acid into the system; that is, cider vinegar, which chemical experiment shows is more nearly allied in its action on the food, to that of the gastric juice, than any other known liquid. Kole Slaw, made by pouring vinegar on crisp, raw cabbage cut up very fine, is digested in about one hour, which is as soon as any other food; while boiled cabbage requires five hours, as also roasted pork. In the Spring of the year when the system becomes feverish and bilious and the blood is thick and impure there is a craving for salads and greens and lettuce, all being used with vinegar; and wise Nature, always watchful and kind, invites the taking of acids by the delicious combination with fruits and berries and tomatoes, as a means of "cooling" the system, which is the result of a more active seperation of the bile from the blood, which scientific experimenters of Paris have ascertained is done through the influence which acids have on the liver, in increasing its activity. Thus it is easy to see that pickles well prepared, with pure cider vinegar, kept in glass jars, hard, green and crisp, not only fully meet the tastes and instincts, but actually promote the heathfulness of the body. Delicate persons and dyspeptics may avoid swallowing the more solid portions of the pickle. To insure that the pickles shall be made free from the poisoning properties of brass and copper vessels, and of the equally destructive ingredients which are used to give them a fresh, green color, that excellent paper, the Baltimore Weekly Commercial, gives as original in its columns the following explicit instructions for making pickles:

PICKLED CUCUMBERS.— Keep the cucumbers in a strong brine for nine days or more; then wash and put them in a bell-metal skillet with lumps of alum through them; cover with vinegar and water. Put on the top to keep in the steam, and place the vessel near the fire that they may become hot, but not so as to boil. When they are a good green put them in glass jars with layers of any spices you may fancy. Fill up the jars with scalding vinegar.

PICKLED ONIONS.— Put the onions in a strong brine for five or six days; then simmer them in equal parts of milk and water; do not let them boil; then take off the fire and dash into very cold water to make them crisp. Place in a jar wlth alternate layers of such spices as suit; then pour on boiled white wine vinegar.

For mixed pickles: Take three dozen large cucumbers sliced; half a peck green tomatoes sliced; half a peck onions peeled and quartered; four large green peppers cut up; one pint small red and green peppers; sprinkle a pint of salt on them. Let them stand over night draining, then add one oz. white pepper; one oz. mace; one oz. white mustard seed; half an oz. cloves; half an oz. celery seed; one oz. tumeric; three table spoonfuls of table mustard; two lbs. sugar; one piece horse- radish. Cover them with the best vinegar and boil all together for half an hour.

FOR PEPPER SAUCE.—Cut the cabbage very fine and salt it to the taste; cut the peppers and onions in thin strips. Take two parts of the cabbage, 1 part onions, and 1 part peppers; season all well, as desired, and cover with boiling vinegar, which may be renewed during the season. A JERSEYMAN says:— The best and surest way of keeping pickles hard and good, is to wash the cucumbers as soon as gathered, and put a layer of fine salt in the bottom of your barrel, and then a layer of cucumbers; then salt again, and so on until the season is over. The water on the cucumbers by washing, is sufficient to make all the brine they need. Keep them covered, and a weight on the cover to keep them closely packed.— Mine keep ln that way three years. When wanted for table use, take them out of the brine, put them in a brass kettle, cover with cold water, and let them stand two or three days; until as green as you would like to have them; then put one teaspoonful of alum and one of saltpetre in the water, and scald in that first water; let them cool in it; then add fresh water, more alum and saltpetre, and scald again; change the water and scald three times, adding alum and saltpetre each time. Cut one of the largest—taste of the inside—if fresh enough drain them, and put in jars. Use the best cider vinegar; spice and pepper to suit your taste Change your vinegar when necessary, and keep from the air.

